# A Curious Case of Acute Glomerulonephritis — Staphylococcus vs Lupus: Case Report and Literature Review

**DOI:** 10.7759/cureus.13138

**Published:** 2021-02-04

**Authors:** Maham A Mehmood, Hitesh Gurjar, Tegveer Sindhu, Asim Haider, Mahnoor Arshad

**Affiliations:** 1 Internal Medicine, BronxCare Health System, New York, USA; 2 Internal Medicine, Faisalabad Medical University, Faisalabad, PAK

**Keywords:** staphylococcus, lupus, hydralazine, kidney biopsy, methylprednisone

## Abstract

Here we report a case of a 65-year-old female, where we encountered acute glomerulonephritis. The patient initially presented with a hemorrhagic blister involving the second through fourth toes with serosanguinous discharge and was on hydralazine for blood pressure control. Differentials included Staphylococcus-associated/lupus nephritis/anti-neutrophil cytoplasmic autoantibody (ANCA) vasculitis. Still, detailed history and meticulous clinical approach with supporting labs and imaging helped us to narrow it to Staphylococcus-associated glomerulonephritis, which is rarely encountered in clinical practice and is associated with high mortality. The management of the patient resulted in a positive outcome and she was discharged home.

## Introduction

Acute glomerulonephritis is often encountered in clinical practice. The differentials are broad and include lupus nephritis, vasculitis, infection-related, and immunoglobulin A (IgA) nephropathy. Good history, physical and meticulous laboratory investigations help narrow down the differentials, as prompt initiation of management can be critical in preventing morbidity and mortality. Staphylococcus-related glomerulonephritis is rare, and early recognition and eradication of infection can be life-saving. 

## Case presentation

A 65-year-old female with medical comorbidities of dementia, diabetes (diet controlled), and hypertension (controlled on hydralazine) presented complaining of progressively worsening left foot ulcer for three days. As per the patient, the ulcer started as a blister and since worsened, accompanied by severe pain. On presentation, her blood pressure was 145/78 mmHg, pulse 71/min, respiratory rate 14/min, temperature 98F. On examination, she was at her baseline mental status (alert, oriented to self and place); her left foot was tender with a hemorrhagic blister involving the second through fourth toes with serosanguinous discharge. The rest of the physical examination was normal. Laboratory examination was noted to have acute kidney injury (blood urea nitrogen (BUN) and creatinine 24 mg/l and 2.4 mg/l respectively), baseline renal function was entirely normal six months prior to presentation, hemoglobin/hematocrit of 10 g/dl and 32% respectively, white blood cell 7 k/ul, erythrocyte sedimentation rate (ESR) 120 mm/hr, C-reactive protein 178 mg/l. Urine analysis was significant for large hematuria and trace proteinuria, urine protein 51 (0-31 mg/dl) and urine creatinine 60 (20-200 mg/dl) (Table 1).

**Table 1:** Autoimmune workup RPR: rapid plasma reagin, RNP: ribonucleoprotein, anti-Sm: anti-Smith, HCV: hepatitis C virus, EIA: enzyme immunoassay

Labs	Patient result	Reference range
Anti-peroxidase	<1	<1AI
Anti Myeloperoxidase	7.6	<1AI
Anti-RNP	<1	<1 AI
Anti-Sm	<1	<1AI
Glomerular basement membrane antibody	<1	<1AI
Histone antibody	9.6	<1U
Anti-DNA AB	5	<4IU/ml
HIV-1/2Ab (EIA)	Negative	Non-reactive
RPR screen	Non-reactive	Non-reactive
Anti-HCV	Negative	Negative

X-ray chest was negative for any acute process. The patient was suspected of having clinical necrotizing fasciitis, she refused computed tomography (CT) of the lower extremity and underwent incision drainage, and debridement and cultures were sent. The patient was started on daptomycin/meropenem/clindamycin and was transferred to the medical floor. Kidney injury was presumably due to prerenal due to underlying infection, that was managed conservatively without dialysis. Antibiotics were deescalated to cefazolin/metronidazole as the cultures from the foot grew methicillin-sensitive *Staphylococcus aureus*. Her post-operative course was complicated with poor foot healing, and the patient ended up getting transmetatarsal amputation and then below-knee amputation (BKA) in a matter of days. Histopathology later reported skin and subcutaneous tissue with acute and chronic inflammation, abscess and granulation tissue. Soft tissue margin and bone margin of resection were unremarkable.

**Figure 1:**
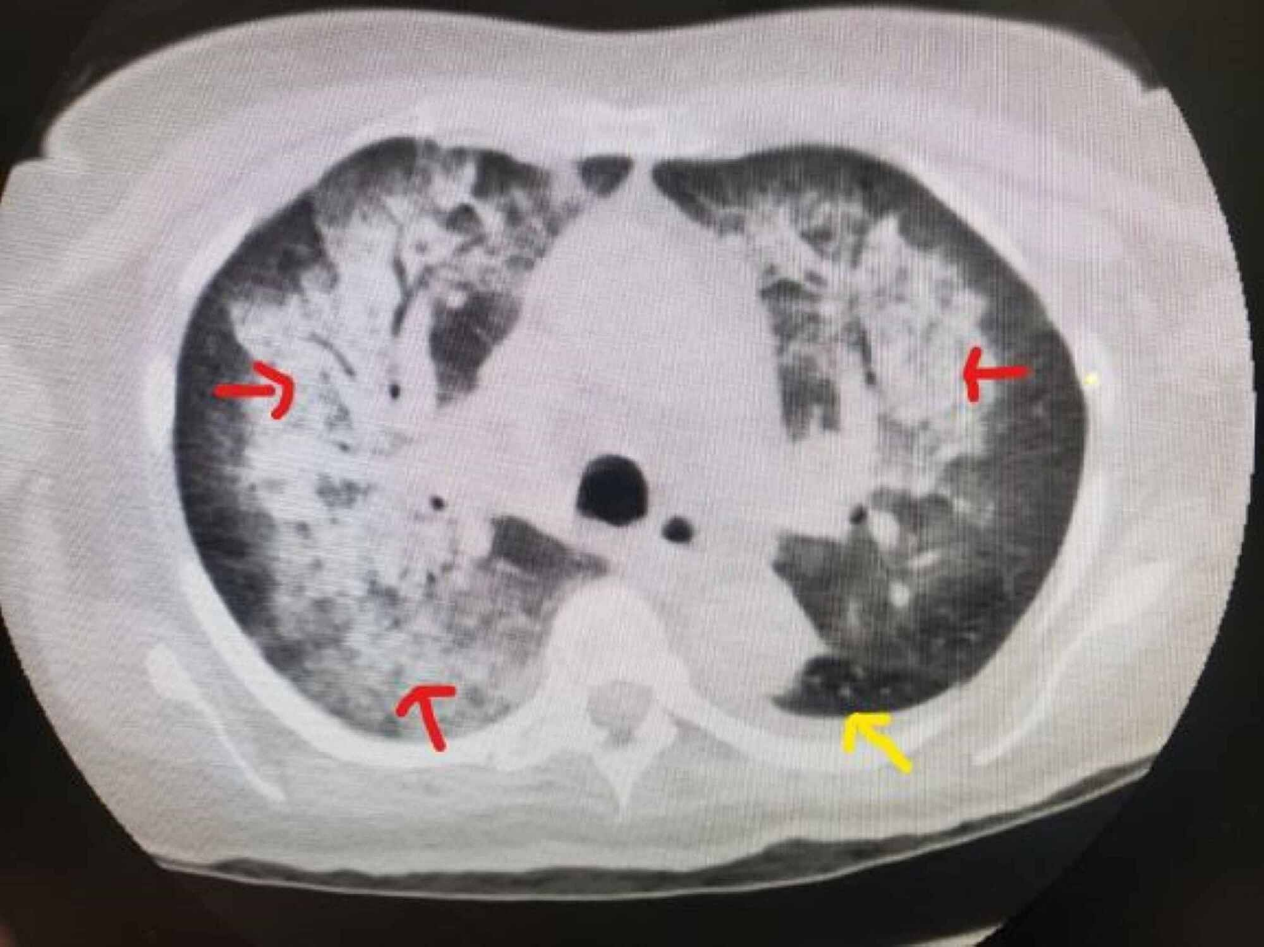
CT chest with diffuse mixed interstitial and alveolar airspace disease (red arrows) and small pleural effusion (yellow arrow)

The renal function continued to worsen, and the creatinine jumped to 4.4 mg/l. The antinuclear antibody was positive with a titer of 1:320, hypocomplementemia (C3 was 53 mg/dl and C4 was 3 mg/dl). At this point, differentials included post-inflammatory glomerulonephritis versus late-onset systemic lupus nephritis versus drug-induced lupus. Hydralazine was placed on hold. Other pertinent labs revealed positive anti-histone antibodies 9.6 unit (ref <0.1), cardiolipin IgM >150 (normal <20) and myeloperoxidase antibodies 9 units and 7.6 units respectively, creatinine jumped up to 6.6 mg/l, anti-DNA, glomerular basement antibody, and antiphospholipid antibody were negative. Hepatitis panel including hepatitis B and C were negative. She also tested negative for HIV. Another differential of anti-neutrophil cytoplasmic autoantibody (ANCA)-associated vasculitis was included, Rheumatology was taken on board, the patient was started on a pulsed dose of methylprednisone, and renal biopsy was planned to differentiate between lupus nephritis/pauci-immune glomerulonephritis. The patient's clinical course took a turn for the worse when she became hypoxic on the floor, was placed on the high flow oxygen, and transferred to the critical care unit. CT chest at this point revealed diffuse mixed interstitial and alveolar airspace disease and small pleural effusions (Figure 1). She also had a 1 g drop in hemoglobin from baseline requiring one packed red blood cell transfusion. She tested negative for coronavirus disease 2019 (COVID-19). 

Suspecting diffuse alveolar hemorrhage, the patient was planned for bronchoscopy, which she and the family adamantly refused. Renal biopsy was suggestive of focal glomerulosclerosis with small mesangial and subepithelial deposits suggestive of membranous lupus nephritis, the resolving phase of infection-related included in the differential. Positive multifocal red blood cell (RBC) casts consistent with the pauci-immune focal crescentic glomerulonephritis associated with ANCA (possibly hydralazine-induced).

The patient had an uneventful course in the critical care unit, completed two weeks course of antibiotics, remained on the tapering doses of prednisone (started on prednisone 60 mg and tapering 10 mg every week), was weaned off oxygen, BKA stump healed well, and creatinine dropped down to 4.4 mg/l and plateaued at 4.0 mg/l without dialysis. The patient was planned to be discharged to short term rehab, but the family refused and decided to take her home. The patient was discharged with improving clinical/lab status on tapering doses of prednisone and outpatient follow up with nephrology/rheumatology/surgery.

Our patient's kidney biopsy showed no definitive findings compatible with vasculitis. Our differentials remained the possibility of post-infectious/systemic lupus erythematosus (SLE)/drug-induced lupus. However, SLE remained lower on the differentials as the anti-DNA antibody and anti-Smith antibody were negative. In our clinical assessment and supporting laboratory examination (Table 2), repeat cardiolipin IgM antibody was normal.

**Table 2:** Improving labs over days ESR: erythrocyte sedimentation rate. CRP: C-reactive protein, BUN: blood urea nitrogen

labs	Day 1	Day 21
ESR (ref 0-30 mmhr)	120	44
CRP (ref <5 mg/l)	178	21
C3 (ref 90-150 mg/dl)	53	92
C4 (ref 16-47 mg/dl)	3	16
BUN (ref 6-20 mg/dl)	105	47
Creatinine (ref 0.5-1.5 mg/dl)	7.2	4.5

We believe that our patient had infectious glomerulonephritis with some element of drug-induced nephritis. Our clinical suspicion was further solidified when the patient showed improvement after below-knee amputation, a continuation of culture-specific antibiotic therapy, and discontinuation of hydralazine.

## Discussion

The differential diagnosis encompassing acute glomerulonephritis is lupus nephritis, vasculitis (such as ANCA-associated vasculitis), and IgA nephropathy, to name a few. Thus, it is prudent to include the antinuclear antibodies, anti-double-stranded DNA antibodies, antineutrophil cytoplasmic antibodies (ANCA), cryoglobulins, hepatitis B and C, HIV, and anti-glomerular basement membrane antibodies in the evaluation. The specific patterns of hypocomplementemia can help narrow down the differentials like in Staphylococcus-associated or other bacterial causes of glomerulonephritis, the typical way is low C3 and normal C4 levels, lupus nephritis is usually associated with reduced levels of both C3 and C4, and mixed cryoglobulinemia is frequently associated with low C4 and normal C3 [1].

Hydralazine is known to be associated with both developing lupus and ANCA-positive vasculitis involving the kidney. The risk for the former is estimated to be 13% [2]. In patients taking hydralazine, anti-histone antibodies are present in 95% [3]. Necrotizing glomerulonephritis is mostly implicated in drug-induced lupus with little or no immune complex deposition [4], although immune complex-mediated glomerulonephritis can occur [5]. The patients with necrotizing glomerulonephritis usually have a perinuclear anti-neutrophil cytoplasmic antibodies (P-ANCA) pattern with anti-myeloperoxidase (MPO) antibodies, plus either anti-lactoferrin or anti-elastase antibodies [4]. This combination of ANCAs is relatively specific for this form of hydralazine-induced vasculitis [6]. The most common symptoms of drug-induced lupus include fever, myalgia, arthralgia, arthritis, rash, and serositis. Hematologic abnormalities, renal disease, and central nervous system involvement, although uncommon, can occur [3].

Staphylococcus-associated glomerulonephritis is rare and occurs more commonly in middle-aged to older adult patients [1]. In one single-center cohort that included 9500 kidney biopsies, the incidence of Staphylococcus-associated glomerulonephritis was found to be 0.8%; the mean age was 55 years, and male to female ratio 3.5:1. Methicillin-resistant *Staphylococcus aureus* (MRSA) and methicillin-sensitive *S. aureus* (MSSA) were implicated in 59 and 27% of cases [7]. The proposed mechanisms of pathogenesis include firstly, glomerular deposition of preformed circulating immune complexes [8], and secondly, Staphylococcal antigens acting as superantigens, activating T cells, polyclonal B-cell and production of polyclonal immunoglobulin A (IgA), immunoglobulin G (IgG), and immunoglobulin M (IgM) [9].

The clinical features in adults with Staphylococcus-associated glomerulonephritis include simultaneous infection with hematuria, proteinuria of varying degrees, a rising serum creatinine, and/or edema [10]. A provisional diagnosis of Staphylococcus-associated glomerulonephritis can be made if the patient has low complement levels, endocapillary proliferation and exudative glomerulonephritis on light microscopy, C3 dominant glomerular staining on immunofluorescence microscopy, and hump-shaped subepithelial deposits on electron microscopy. Kidney biopsy is definitive, which typically shows hump-shaped subepithelial electron-dense deposits [1]. Further confirmatory is the resolution of the disease activity after eradication of the infection.

Eliminating infections is the cornerstone of the management of Staphylococcus-associated glomerulonephritis; rest involves relieving symptoms and controlling hypertension and edema by limiting salt intake and using diuretics. The role of immunosuppressants in treating Staphylococcus-associated glomerulonephritis is not well understood and is controversial in patients with an active infection, as giving high-dose glucocorticoids can lead to worsening of the condition or death [11]. Nasr et al. observed no correlation between glucocorticoid therapy and renal outcomes. In a series of patients with Staphylococcus-associated glomerulonephritis, 24% had complete renal function recovery, 32% had persistent renal dysfunction, and 44% progressed to end-stage renal disease [10].

We introduced steroids in our patient because there was an element of drug-induced lupus, and our experience had a positive outcome. The long-term renal prognosis in patients with Staphylococcus-associated glomerulonephritis is relatively low, which may be related to the fact that affected patients are usually older and have comorbidities such as diabetes.

## Conclusions

Clinicians, when faced with acute glomerulonephritis, should keep their eyes open for any possible infection as an inciting factor and, at the same time, rule out other important causes as well. The successful management lies in early recognition, eradicating the inciting element, and prompt initiation of a specific therapy. Where the diagnosis is mixed, like in our case, clinicians must weigh the benefits versus risk of starting immunosuppressive therapy like steroids, as the steroids can act as a double-edged sword, so their judicious use is recommended.
